# Plasma C24:0- and C26:0-lysophosphatidylcholines are reliable biomarkers for the diagnosis of peroxisomal β-oxidation disorders

**DOI:** 10.1016/j.jlr.2024.100516

**Published:** 2024-02-04

**Authors:** Blai Morales-Romero, José Manuel González de Aledo-Castillo, Cristina Fernández Sierra, Carmen Martínez Carreira, Carles Zaragoza Bonet, Rosa Fernández Bonifacio, Maria Antònia Caro Miró, Ana Argudo-Ramírez, Rosa María López Galera, Judit García-Villoria

**Affiliations:** 1 Section of Inborn Errors of Metabolism-IBC, Biochemistry and Molecular Genetics Department, Hospital Clínic de Barcelona, Barcelona, Spain; 2 Biomedical Research Institute August Pi i Sunyer (IDIBAPS), Barcelona, Spain; 3 Center for Biomedical Research Network on Rare Diseases (CIBERER), Madrid, Spain; 4 CORE Laboratory, Biochemistry and Molecular Genetics Department, Hospital Clínic de Barcelona, Barcelona, Spain

**Keywords:** lipids, lysophospholipid, lipids/oxidation, fatty acid/transport, VLCFA, tandem mass spectrometry, Zellweger spectrum disorders, Zellweger syndrome, Adrenoleukodystrophy, ALD female

## Abstract

The gold-standard diagnostic test for peroxisomal disorders (PDs) is plasma concentration analysis of very long-chain fatty acids (VLCFAs). However, this method’s time-consuming nature and limitations in cases which present normal VLCFA levels necessitates alternative approaches. The analysis of C26:0-lysophosphatydylcholine (C26:0-LPC) in dried blood spot samples by tandem-mass spectrometry (MS/MS) has successfully been implemented in certain newborn screening programs to diagnose X-linked adrenoleukodystrophy (ALD). However, the diagnostic potential of very long-chain LPCs concentrations in plasma remains poorly understood. This study sought to evaluate the diagnostic performance of C26:0-LPC and other very long-chain LPCs, comparing them to VLCFA analysis in plasma. The study, which included 330 individuals affected by a peroxisomal β-oxidation deficiency and 407 control individuals, revealed that C26:0- and C24:0-LPC concentrations demonstrated the highest diagnostic accuracy (98.8% and 98.4%, respectively), outperforming VLCFA when C26:0/C22:0 and C24:0/C22:0 ratios were combined (98.1%). Combining C24:0- and C26:0-LPC gave the highest sensitivity (99.7%), with ALD females exhibiting notably higher sensitivity compared with the VLCFA ratio combination (98.7% vs. 93.5%, respectively). In contrast, C22:0-LPC exhibited suboptimal performance, primarily due to its low sensitivity (75%), but we identified a potential use to help distinguish between ALD and Zellweger spectrum disorders. In summary, MS/MS analysis of plasma C24:0- and C26:0-LPC concentrations represents a rapid and straightforward approach to diagnose PDs, demonstrating superior diagnostic accuracy, particularly in ALD females, compared with conventional VLCFA biomarkers. We strongly recommend integrating very-long chain LPC plasma analysis in the diagnostic evaluation of individuals suspected of having a PD.

Peroxisomal disorders (PDs) constitute a diverse group of genetic diseases characterized by deficiencies in proteins responsible for essential metabolic functions, including the β-oxidation of very long-chain fatty acids (VLCFAs), the α-oxidation of phytanic acid and the biosynthesis of plasmalogens, among others (1).

PDs can be categorized into two main groups: single enzyme deficiencies and peroxisome biogenesis disorders (PBDs). In the first group, enzyme defects primarily affect specific metabolic pathways. The most prevalent example of this is X-linked adrenoleukodystrophy (ALD), with an incidence rate of 1/17,000. ALD is caused by a deficiency in the import of VLCFA into the peroxisome due to mutations in the *ABCD1* gene. It is known for its variable clinical presentation in males, which can range from adrenal insufficiency to rapidly progressive and fatal cerebral demyelination (cerebral ALD) (2, 3). Additionally, 80% of heterozygous ALD females present with progressive myelopathy (2, 4).

The second PD group (comprising PBD) gives rise to Zellweger spectrum disorders (ZSDs). This group includes a set of 13 diseases that result from mutations in the different *PEX* genes coding for peroxin proteins involved in peroxisome assembly and maintenance. These result in the disruption of multiple metabolic pathways. These disorders exhibit a broad clinical presentation that ranges from severe and lethal neonatal forms characterized by symptoms such as severe hypotonia, seizures, hepatocellular dysfunction, and facial dysmorphia to milder presentations in adulthood, which can include sensorineural hearing loss, retinopathy, and amelogenesis imperfecta (Heimler syndrome) or cerebellar ataxia and peripheral neuropathy (5, 6, 7).

Both ZSD and ALD, along with other deficiencies, lead to the accumulation of VLCFA due to impaired β-oxidation. This impairment is reflected in elevated plasma levels of C26:0 and an increase of the C24:0/C22:0 and C26:0/C22:0 concentration ratios. VLCFA quantification through direct transesterification (DT) and gas chromatography (GC) analysis has demonstrated excellent diagnostic performance for decades and remains the gold-standard method for diagnosing peroxisomal β-oxidation deficiencies (8, 9, 10).

However, it is important to note that VLCFA analysis through DT and GC presents significant limitations, including time-consuming and labor-intensive sample processing and analysis. Moreover, nonspecific alterations may be observed under various conditions, such as hemolyzed and postprandial samples, patients with hyperlipidemia, severe liver disease, diabetic ketoacidosis, or in samples from patients on ketogenic diets (11). Furthermore, between 15% and 30% of heterozygous ALD females, as well as some patients with a mild ZSD phenotype, exhibit normal VLCFA concentrations (4, 7, 9), making the diagnosis challenging in these specific subgroups. Consequently, there remains ample room for improvement in the biochemical diagnosis of these conditions by identifying more sensitive and specific biomarkers and developing simpler and faster analytical methods.

In recent years, the efficacy of hematopoietic stem cell transplantation as an early intervention for patients with cerebral ALD (12, 13) has sparked interest in the search for novel biomarkers suitable for analysis through liquid chromatography and tandem-mass spectrometry (LC-MS/MS). Analytical methods based on this technology are quicker and require simpler sample processing compared with DT and GC, making them suitable for newborn screening purposes. Hubbard and collaborators demonstrated that lysophosphatidylcholine (LPC) containing hexacosanoic acid (C26:0-LPC) was elevated in dried blood spot (DBS) samples in both male ALD and ZSD patients. They went on to develop the first LC-MS/MS method for its quantification (14, 15). Since then, the analysis of C26:0-LPC in DBS samples using LC-MS/MS has been implemented in various newborn screening programs, proving highly effective in identifying both ALD and ZSD patients (16, 17, 18, 19, 20, 21, 22, 23, 24, 25).

In addition, several studies have been conducted in various countries to evaluate the effectiveness of C26:0-LPC in DBS samples for detecting ALD in newborns, consistently demonstrating reliable performance (26, 27, 28, 29, 30, 31, 32). It has even been suggested that C26:0-LPC in DBS samples might be a more sensitive biomarker for the detection of heterozygote ALD females compared with current plasma VLCFA analysis (26).

Despite the benefits of DBS C26:0-LPC analysis in the newborn screening setting, there is limited knowledge about the general diagnostic performance using plasma concentrations of C26:0-LPC in patients suspected of having a PD. In this context, Jaspers *et al.* assessed the diagnostic reliability of plasma concentrations of C26:0-LPC in ALD and ZSD patients, reporting a diagnostic accuracy of 100% (28). However, the cohort study was limited to just 80 patients and 67 controls, with only 19 ALD females included. Therefore, larger cohort studies are needed to more comprehensively evaluate the diagnostic performance of C26:0-LPC for PDs.

In this study, after first validating a method to measure very long-chain LPCs in plasma, our primary objective was to evaluate the diagnostic accuracy of using plasma concentrations of very long-chain LPCs in a large cohort of 340 patients affected by a peroxisomal β-oxidation deficiency, including 77 ALD females, and in a set of 407 control individuals. In addition, our secondary objective was to compare the diagnostic performance of plasma very long-chain LPCs with VLCFA gold-standard analysis. Furthermore, we wanted to explore the clinical utility of measuring multiple LPCs in plasma (including C22:0- and C24:0-LPC) for diagnosing PDs.

## Materials and Methods

### Cohort study and sample collection

The study included plasma samples from 340 individuals diagnosed with a peroxisomal β-oxidation deficiency. Diagnosis was confirmed either through molecular studies or the detection of altered specific biomarkers (plasma VLCFA, phytanic and pristanic acids, and/or erythrocyte plasmalogens) coupled with a compatible clinical presentation. Among the individuals included in the study, molecular diagnosis data were available for 32% of ALD males (50/155), 80% of ALD females (59/74), and 30% of ZSD patients (18/61). For females clinically suspected of ALD without available molecular diagnosis data, only those with elevated C26:0 levels and both C26:0/C22:0 and C24:0/C22:0 ratios were included in this study to ensure diagnosis certainty from a biochemical perspective. Additionally, the study included 47 unclassified patients exhibiting an altered VLCFA profile, characterized by elevated C26:0 levels and elevated C26:0/C22:0 and C24:0/C22:0 ratios, but for whom no clinical or molecular data were available for definitive diagnosis. Furthermore, we analyzed 498 plasma samples from control individuals to establish cutoff values. An additional 407 control plasma samples were examined to assess the diagnostic accuracy of very long-chain LPCs. All control samples were from individuals for whom our laboratory had excluded the presence of a peroxisomal β-oxidation deficiency and other inherited metabolic disorders.

All samples were obtained in accordance with the principles outlined in the Declaration of Helsinki. Approval of the Research Ethics Committee was not required, since all measurements were performed as part of routine diagnostic procedures, and data were anonymized for subsequent analysis. Some plasma samples from individuals affected by PDs were sourced from our sample repository of inherited metabolic disorders in line with the appropriate ethical approval (ISCIII, R090618-008).

### Plasma VLCFA analysis

Plasma quantification of VLCFA was performed by analyzing the corresponding fatty acid methyl-esters of C22:0, C24:0, and C26:0 following a previously described method (8). In brief, 8 nmol of the C19:0 internal standard were added to 50 μl of plasma. DT was achieved by adding 2 ml of methanol-toluene (4:1, v/v) and 0.2 ml of acetyl chloride, followed by incubation at 100ºC in an oven for 1 h. Subsequently, 5 ml of aqueous 6% potassium carbonate were added to stop the reaction and neutralize the mixture. After thorough mixing and centrifugation, the upper organic layer was collected and evaporated under nitrogen. The residue was then reconstituted in 200 μl of hexane and transferred to an injection vial. Next, 1 μl was injected into a 6890 GC system (Agilent Technologies, Santa Clara, CA) equipped with a 60m BPX70 GC Capillary Column (Trajan Scientific and Medical, Bethel, CT) and a flame-ionization detector. VLCFA levels were quantified using standard calibration curves in Agilent OpenLab Software (version 3.2, Agilent Technologies, Santa Clara, CA). The cutoff values for VLCFA analysis were those routinely employed in our laboratory, determined by the 97.5^th^ percentile of control individuals.

### Validation of the LC-MS/MS analytical method for plasma very long-chain LPCs

Plasma very long-chain LPCs were analyzed by adapting the NeoBase™ 2 Non-derivatized MSMS kit (PerkinElmer, Waltham, MA). A validation of the method was conducted for plasma samples in accordance with the EU Regulation 2017/746 on In Vitro Diagnostic Medical Devices for in-house developed tests (33). The assay’s linearity was established by preparing nine standard samples with concentrations ranging from 0.0031 to 8 μmol/L for C22:0-, C24:0-, and C26:0-LPC (Avanti Polar Lipids, Alabaster, AL). These standard samples were processed in the same manner as the study samples.

Calibration curves were generated through linear regression analysis of the area ratio between each very long-chain LPC calibrator and the internal standard (^2^H4-C26:0-LPC), along with the standard concentrations. Quantification (limit of quantification) and detection (limit of detection) limits were determined by analyzing successive dilutions of C22:0-, C24:0-, and C26:0-LPC standards until reaching signal-to-noise ratios of ≥3 and ≥10, respectively.

Method precision was assessed by calculating the intraassay coefficient of variation (CV) from 10 analyses of a pooled sample conducted on the same day. Interassay variability was determined by calculating the CV from the analysis of another pooled sample independently processed on 10 different days.

Recovery was evaluated by adding known amounts of C22:0-, C24:0-, and C26:0-LPC standards (0.125 μmol/L, 0.25 μmol/L, and 0.50 μmol/L) to a pooled sample. All samples were analyzed in six replicates, and recovery was calculated by comparing the calculated concentrations with those obtained from the analysis of three methanol samples containing the same quantity of standards (without matrix).

The matrix effect was evaluated by adding known amounts of C22:0-, C24:0-, and C26:0-LPC standards (0.125 μmol/L, 0.25 μmol/L, and 0.50 μmol/L) to six different plasma samples and comparing the recoveries with those obtained in three methanol samples containing the same quantity of standards (without matrix).

The reference interval for each LPC was defined as the value range between the 2.5^th^ and the 97.5^th^ percentiles obtained by analyzing 498 plasma control samples, comprising 276 males and 222 females. The upper limit cutoffs were set at above the 97.5^th^ percentile for each measured LPC.

Finally, considering that hypertriglyceridemia is a known common cause of false positive results in plasma VLCFA analysis, we also assessed the specificity of very long-chain LPCs using 20 samples obtained from patients with elevated triglyceride levels due to dyslipidemia. Triglyceride concentrations ranged between 5.65 and 29.62 mmol/L (median = 12.08 mmol/L, cutoff <1.69 mmol/L).

### Plasma very long-chain LPCs analysis

Very long-chain LPCs (C22:0-, C24:0-, and C26:0-LPC) were analyzed in all patient and control individuals using tandem mass spectrometry (MS/MS) with a Xevo TQD analyzer (Waters Corporation, Milford, MA) and the NeoBase™ 2 Non-derivatized MSMS kit (PerkinElmer, Waltham, MA,), following the manufacturer’s protocol with minor adaptations for plasma analysis. This kit simultaneously quantifies C26:0-LPC, amino acids, acylcarnitines, and succinylacetone based on the method previously described by Haynes *et al.* 2016 (34). In brief, 3 μl of plasma were applied to a 3.2 mm spot of Whatman 903 filter paper (Whatman International Ltd, Kent, UK). Analytes were extracted by adding 125 μl of the extraction working solution containing ^2^H4-C26:0-LPC as internal standard and then shaking at 45°C for 45 min. Following extraction, 5 μl of the supernatant were directly injected into Xevo TQD analyzer using both positive ionization and multiple reaction monitoring modes.

### Statistical analysis

Statistical analysis was conducted using GraphPad Prism 9 software (version 9.0.2, GraphPad Software, Boston, MA). Concentrations of very long-chain LPCs and VLCFA were reported as median values with corresponding ranges. Group comparisons were made using the *t*-student or Mann-Whitney *U* test as appropriate, with *P*-values <0.05 considered statistically significant. Standard measures of diagnostic accuracy, including sensitivity, specificity, and positive predictive values, were computed for all biomarkers. Receiver operating characteristic curves were generated to assess the performance of selected biomarkers in differential diagnosis. For visual representation, data were presented in box-and-whisker format, where the central band within the box represented the median, and the lower and upper borders of the box corresponded to the 25^th^ (Q1) and the 75^th^ (Q3) percentiles of the distribution, respectively. The lower and upper fences were calculated using the Tukey formula (Q1 − 1.5 × IQR; Q3 + 1.5 × IQR), where IQR stands for the interquartile range. To assess specificity in samples with hypertriglyceridemia, concentration values for very long-chain LPCs and VLCFA were displayed using scatterplots.

## Results

### The LC-MS/MS method for analyzing plasma very long-chain LPCs demonstrated reliable performance

As previously mentioned, the quantification of very long-chain LPCs using the NeoBase™ 2 Non-derivatized MSMS kit for DBS samples was adapted for plasma analysis and subsequently validated. The results from the method validation are summarized in Table 1. A satisfactory performance was achieved for all three biomarkers (C22:0-, C24:0-, and C26:0-LPC). Linearity of the assay was demonstrated within the concentration range of 0.06 and 8.00 μmol/L, with coefficient of linear regression (*r*^2^) values ranging from 0.993 to 0.998 (SD < 0.003) for all three very long-chain LPCs. The mean intraassay and interassay CV were both ≤22% for all three analytes. Additionally, C24:0- and C26:0-LPC exhibited good recovery and demonstrated no matrix effects, while C22:0-LPC showed slightly poorer performance in both parameters.

**Table 1:** Validation parameters for plasma C22:0-, C24:0-, and C26:0-LPC analysis using LC-MS/MS

Compound	Linearity Range (μmol/L)	LOD (μmol/L)	LOQ (μmol/L)	Intraassay CV (%)	Interassay CV (%)	Recovery (%)^a^			Matrix Effect (%)^a^			Cutoff Value (μmol/L)^b^
						L	M	H	L	M	H	
C22:0-LPC	0.06–8.00	0.015	0.06	17	22	85	86	68	92	101	72	0.19
C24:0-LPC	0.06–8.00	0.015	0.06	22	22	98	104	70	95	104	78	0.27
C26:0-LPC	0.07–8.00	0.015	0.07	19	22	93	97	73	108	100	88	0.17

LOD, limit of detection; LOQ, limit of quantification; LPC, lysophosphatidylcholine.

a Very long-chain LPC concentrations were as follows: L (low) = 0.125 μmol/L, M (medium) = 0.25 μmol/L, and H (high) = 0.50 μmol/L).

b The cutoff values were calculated as the 97.5^th^ percentile of 498 samples from control individuals.

Cutoff values for plasma very long-chain LPCs were determined by analyzing plasma samples from 498 control individuals (Table 2). Differences in LPC concentrations among various age groups were examined within this cohort (supplemental Fig. S1). Notably, no differences in C26:0-LPC concentrations were observed, while C22:0- and C24:0-LPC concentrations were found to be significantly higher in patients aged between 1 month and 1 year when compared with other age groups. Furthermore, newborns (≤1 month of age) exhibited significantly lower plasma C22:0-LPC concentrations. Sex-specific variations in very long-chain LPC concentrations were also assessed, and no differences were observed between males and females (supplemental Fig. S2).

**Table 2:** Plasma concentrations of very long-chain LPCs and VLCFA in patients and control individuals

Parameter (Units)	Controls (N = 407)	All Patients (N = 340)	ALD Males (N = 155)	ALD Females (N = 74)	ZSD (N = 61)	UnPD^a^ (N = 47)
C26:0-LPC (μmol/L)	0.08 (0.01–0.25)	0.56 (0.06–3.68)	0.58 (0.18–1.84)	0.38 (0.06–1.00)	1.43 (0.16–3.68)	0.61 (0.29–2.08)
C24:0-LPC (μmol/L)	0.14 (0.00–0.32)	0.69 (0.11–2.17)	0.76 (0.28–2.00)	0.48 (0.11–1.32)	0.73 (0.28–2.17)	0.65 (0.36–1.20)
C22:0-LPC (μmol/L)	0.10 (0.00–0.25)	0.29 (0.07–1.23)	0.36 (0.08–1.23)	0.24 (0.07–0.93)	0.19 (0.07–0.57)	0.23 (0.12–0.66)
C20:0-LPC (μmol/L)	0.18 (0.00–0.54)	0.43 (0.08–2.13)	0.52 (0.08–1.80)	0.40 (0.13–1.82)	0.42 (0.08–2.13)	0.31 (0.15–1.30)
C26:0 (μmol/L)	0.65 (0.19–1.59)	2.81 (0.12–23.00)	3.00 (1.29–8.05)	1.92 (0.12–3.90)	6.74 (0.77–23.00)	2.80 (1.36–9.30)
C24:0/C22:0	0.83 (0.37–1.25)	1.56 (0.09–3.02)	1.60 (1.06–2.25)	1.25 (0.09–1.80)	1.66 (0.91–3.02)	1.67 (1.31–2.28)
C26:0/C22:0	0.010 (0.000–0.071)	0.058 (0.001–0.810)	0.060 (0.029–0.170)	0.031 (0.001–0.069)	0.257 (0.023–0.810)	0.058 (0.033–0.484)
Age (years)	7.5 (0.00–79.1)	14.6 (0.0–78.3)	21.9 (1.6–67.0)	41.0 (2.3–78.3)	0.2 (0.0–10.2)	8.6 (0.5–28.3)

N, number of patients/controls; ALD, X-linked adrenoleukodystrophy; ZSD, Zellweger spectrum disorders patients; UnPD, unclassified peroxisomal disorder patients.

Results are expressed as medians, with ranges displayed in brackets. The median ages of individuals and their respective ranges are also provided for each patient/control group.

a Patients presenting with peroxisomal β-oxidation deficiency, as indicated by elevated C26:0 levels and both C26:0/C22:0 and C24:0/C22:0 ratios in VLCFA analysis, without a definitive differential diagnosis.

### Plasma C26:0- and C24:0-LPC exhibited higher diagnostic accuracy than plasma VLCFA

**Fig. 1:**
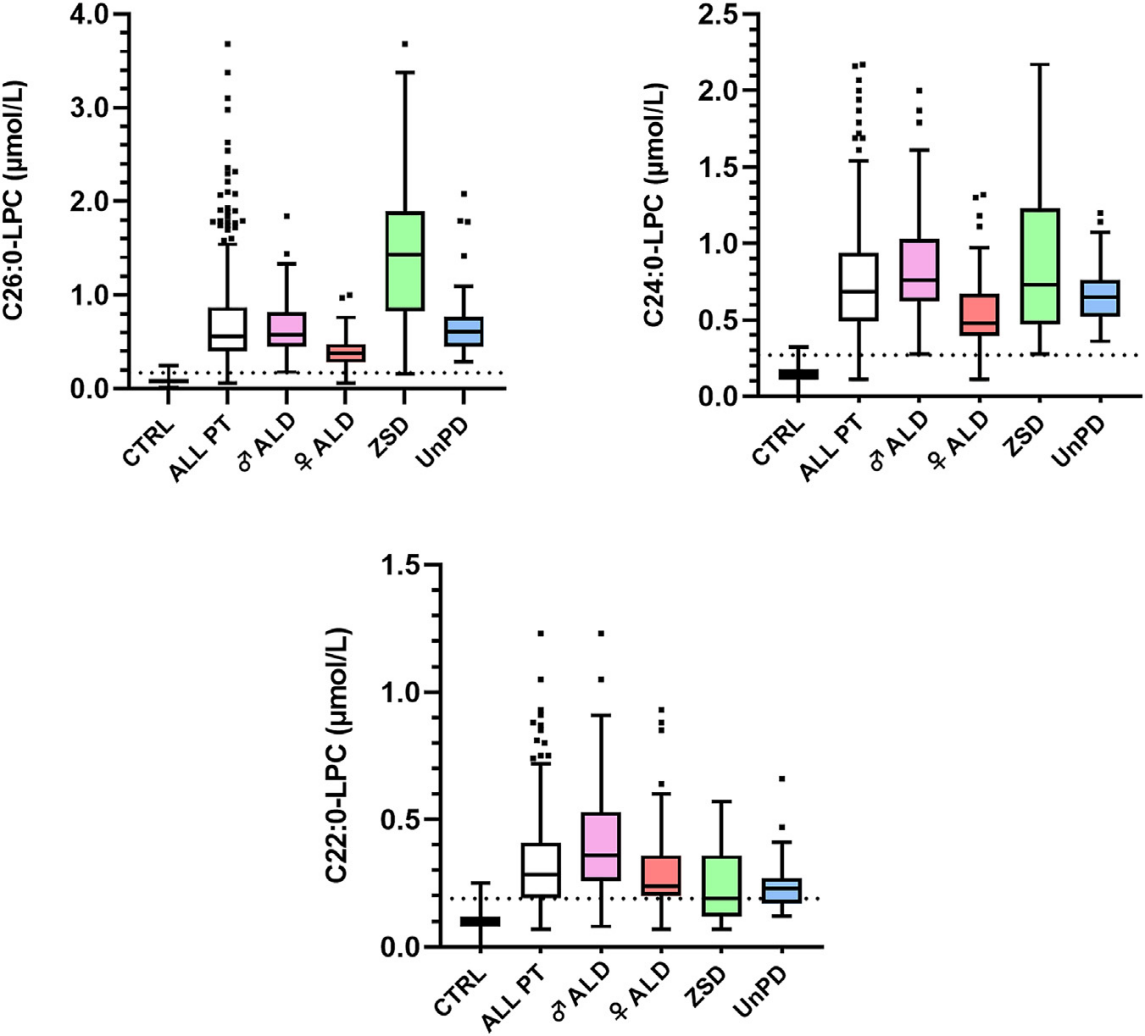
Plasma C24:0- and C26:0-LPC showed excellent diagnostic accuracy for peroxisomal β-oxidation deficiencies. Plasma concentrations of very long-chain LPCs were analyzed in 340 patients and 407 control individuals. Each graph corresponds to one analyte (C26:0-, C24:0-, and C22:0-LPC). Patients are presented both as a collective group and divided by disease category. Plasma LPCs concentrations are plotted in box-and-whisker format, with dotted lines indicating the cutoff values (C26:0-LPC ≤ 0.17 μmol/L, C24:0-LPC ≤ 0.27 μmol/L, and C22:0-LPC ≤ 0.19 μmol/L). Abbreviations are as follows, with the number of patients in each group shown in brackets: CTRL = controls (n = 407), ALL PT = total patients (n = 340), ♂ ALD = male adrenoleukodystrophy patients (n = 155), ♀ALD = female adrenoleukodystrophy patients (n = 77), ZSD = Zellweger spectrum disorder patients (n = 61), UnPD = Patients with a deficient peroxisomal β-oxidation by VLCFA analysis lacking a differential diagnosis (n = 47). Significantly higher concentrations of all three very long-chain LPCs were observed in all disease groups when compared with control individuals (*P* < 0.0001). The highest C24:0- and C26:0-LPC concentrations were observed in ZSD patients (ranging from 0.28 to 2.17 μmol/L and 0.16–3.68 μmol/L, respectively), whereas ALD males presented the highest C22:0-LPC values (ranging from 0.08 to 1.23 μmol/L). ALD males also exhibited significantly higher concentrations of C22:0-LPC (*P* < 0.0001), C24:0-LPC (*P* < 0.0001), and C26:0-LPC (*P* ≤ 0.001) than ALD females. Plasma C26:0-LPC concentrations were significantly higher in ZSD patients compared with ALD males (*P* < 0.0001), while an inverse relation was observed for plasma C22:0-LPC (*P* < 0.0001). No differences were observed for plasma C24:0-LPC between these two groups.

Plasma concentrations of very long-chain LPCs and VLCFA were measured in samples obtained from 340 patients and 407 control individuals, as detailed in Table 2. The concentrations of C22:0-, C24:0-, and C26:0-LPC are also graphically represented in Figure 1. In all disease groups, significantly higher concentrations of all three very long-chain LPCs were observed when compared with control individuals (*P* < 0.0001).

The diagnostic performance of both plasma very long-chain LPCs and VLCFA was further evaluated by considering each parameter individually and in combination, as summarized in Table 3. Regarding VLCFA analysis, the combination of C26:0 and C24:0/C22:0 and C26:0/C22:0 ratios, with a positive result defined as an increase in at least one of the measurements, showed the highest sensitivity (98.8%), although specificity was relatively low (95.6%) due to a high rate of false-positive results for C26:0 analysis.

**Table 3:** Diagnostic performance of plasma analysis for very long-chain LPC and VLCFA

Analyte Class	Biomarker	Cutoff Value	All PT (N = 340)	ALD Females (N = 77)	ALD Males (N = 155)	ZSD (N = 61)	UnPD^a^ (N = 47)	CTRL (N = 407)	Overall Diagnostic Accuracy^b^ (%)
			Overall Sensitivity (%) (FN)	Sensitivity (%) (FN)	Sensitivity (%) (FN)	Sensitivity (%) (FN)	Sensitivity (%) (FN)	Specificity (%) (FP)	
LPC	C26:0-LPC	0.17 μmol/L	98.8 (4)	96.1 (3)	**100.0 (0)**	98.4 (1)	**100.0 (0)**	**98.8 (5)**	**98.8**
LPC	C24:0-LPC	0.27 μmol/L	98.5 (5)	93.5 (5)	**100.0 (0)**	**100.0 (0)**	**100.0 (0)**	98.3 (7)	98.4
LPC	C26:0-LPC ∨ C24:0-LPC	—	**99.7 (1)**	**98.7 (1)**	**100.0 (0)**	**100.0 (0)**	**100.0 (0)**	97.3 (11)	98.4
LPC	C26:0-LPC ∨ C22:0-LPC	—	99.4 (2)	**98.7 (1)**	**100.0 (0)**	98.4 (1)	**100.0 (0)**	97.1 (12)	98.1
VLCFA	C24:0/C22:0 ∨ C26:0/C22:0	—	98.2 (6)	93.5 (5)	**100.0 (0)**	98.4 (1)	**100.0 (0)**	98.0 (8)	98.1
LPC	C24:0-LPC ∨ C22:0-LPC	—	98.5 (5)	93.5 (5)	**100.0 (0)**	**100.0 (0)**	**100.0 (0)**	97.1 (12)	97.7
VLCFA	C26:0 ∨ C26:0/C22:0	—	98.2 (6)	93.5 (5)	**100.0 (0)**	98.4 (1)	**100.0 (0)**	96.3 (15)	97.2
VLCFA	C26:0 ∨ C24:0/C22:0 ∨ C26:0/C22:0	—	98.8 (4)	96.1 (3)	**100.0 (0)**	98.4 (1)	**100.0 (0)**	95.6 (18)	97.1
VLCFA	C26:0/C22:0	0.025	94.7 (18)	77.9 (17)	**100.0 (0)**	98.4 (1)	**100.0 (0)**	98.8 (5)	96.9
VLCFA	C26:0	1.12 μmol/L	97.6 (8)	90.9 (7)	**100.0 (0)**	98.4 (1)	**100.0 (0)**	96.8 (13)	97.2
VLCFA	C24:0/C22:0	1.04	96.5 (12)	90.9 (7)	**100.0 (0)**	91.8 (5)	**100.0 (0)**	98.5 (6)	97.6
LPC	C22:0-LPC	0.19 μmol/L	75.0 (85)	77.9 (17)	89.0 (17)	44.3 (34)	63.8 (17)	98.0 (8)	87.6

VLCFA, very-long chain fatty acids; LPCs, very long-chain lysophosphatidylcholines; N, number of individuals; ∨, and/or (increase of at least one magnitude); ALL PT, all patients; ALD, X-linked adrenoleukodystrophy; ZSD, Zellweger spectrum disorder patients; UnPD, unclassified peroxisomal disorder patients; CTRL, control individuals; TP, true positives, number of patients correctly classified; FN, false negatives, number of patients wrongly classified; FP, false positives, number of control individuals wrongly classified; TN, true negatives, number of control individuals correctly classified.

Biomarkers are arranged in descending order of diagnostic accuracy. The highest sensitivity and specificity values for each column (patient group) are highlighted in bold. Results corresponding to the combinations of C26:0- and C24:0-LPC, as well as the C24:0/C22:0 and C26:0/C22:0, used in this study to compare the diagnostic performance of LPCs and VLCFA, are shaded in gray cells.

a Patients presenting with peroxisomal β-oxidation deficiency, as indicated by elevated C26:0 levels and both C26:0/C22:0 and C24:0/C22:0 ratios in VLCFA analysis, without a specific differential diagnosis.

b Proportion of correctly classified individuals = (TP/FN)/(FP/TN).

However, the combination of C24:0/C22:0 and C26:0/C22:0 exhibited the best diagnostic accuracy for VLCFA analysis (98.1%). For this reason, this combination was selected for comparison with the performance of very long-chain LPCs. In this regard, C26:0- and C24:0-LPC demonstrated sensitivities of 98.8% and 98.5% and specificities of 98.8% and 98.3%, respectively, higher than those observed for the combination of C24:0/C22:0 and C26:0/C22:0.

Furthermore, the combination of C24:0- and C26:0-LPC exhibited the highest sensitivity (99.7%) among all the evaluated parameters and their different combinations, along with good specificity (97.3%). Consequently, plasma C24:0- and C26:0-LPC showed better performance compared with the plasma C24:0/C22:0 and C26:0/C22:0 combination, demonstrating higher diagnostic accuracies both individually and in combination. Conversely, C22:0-LPC analysis showed the poorest performance, with a diagnostic accuracy of 87.6% and a sensitivity and a specificity of 75.0% and 98.0%, respectively.

Considering the different disease groups, both the combinations of C26:0/C22:0 with C24:0/C22:0 ratios and C24:0-LPC with C26:0-LPC demonstrated a sensitivity of 100.0% in ALD males and unclassified patients. However, for ALD females and ZSD patients, the combination of C24:0- and C26:0-LPC exhibited higher sensitivities (98.7% and 100.0%, respectively) than the C26:0/C22:0 and C24:0/C22:0 ratio combination (96.1% and 98.4%, respectively). In this context, it is worth noting that patients who were misclassified by C26:0-LPC analysis included three out of 77 ALD females, with C26:0-LPC concentrations of 0.06, 0.15, and 0.17 μmol/L (cutoff ≤ 0.17 μmol/L), and one out of 61 ZSD with Heimler syndrome, the mildest phenotype of the ZSD, showing a C26:0-LPC concentration of 0.16 μmol/L.

Regarding C24:0-LPC analysis, 5 out of 77 ALD females exhibited normal plasma concentrations of 0.11, 0.21, 0.27, 0.24, and 0.26 μmol/L (cutoff ≤0.27 μmol/L), whereas the Heimler syndrome patient in this case had a slight increase in C24:0-LPC (0.28 μmol/L). In most cases, these individuals showed concentrations close to or exactly at the cutoff values, and nearly all of them were correctly classified when C24:0- and C26:0-LPC were considered in combination. However, there was one exception, an ALD woman who presented normal C24:0- and C26:0-LPC concentrations (0.11 and 0.06 μmol/L, respectively), as well as normal VLCFA levels.

Regarding VLCFA analysis, five out of 77 ALD females presented negative results when the C26:0/C22:0 with C24:0/C22:0 ratio combination was considered, four of whom had an altered LPCs analysis. The patient with Heimler syndrome (one out of 61 ZSD patients) also presented normal VLCFA levels.

The highest positive predictive value (PPV) for very long-chain LPCs analysis was achieved when all three analytes were elevated (100.0%), while an increase in C24:0- and C26:0-LPC with normal C22:0-LPC concentration resulted in only a slightly lower PPV (99.7%). Similarly, for VLCFA analysis, when C26:0 and both C26:0/C22:0 and C24:0/C22:0 ratios were increased, the PPV was 99.4%.

**Fig. 2:**
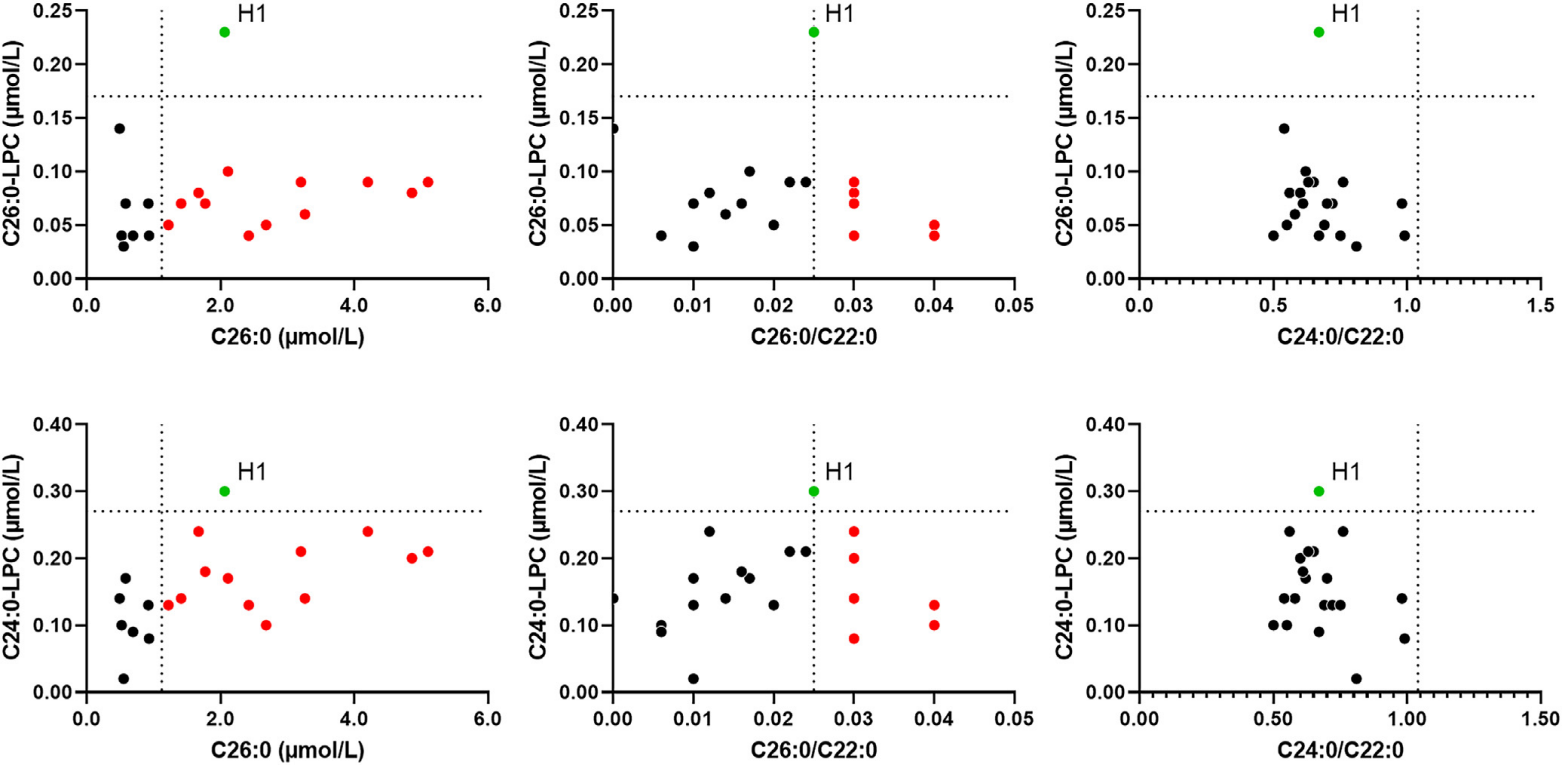
C24:0- and C26:0-LPC showed higher specificity than VLCFA in plasma samples with elevated triglyceride concentrations. Plasma very long-chain LPCs and VLCFA were analyzed in samples collected from individuals with hypertriglyceridemia. Each data point on the scatter plot represents a plasma sample obtained from a single individual (n = 20; median triglyceride concentration = 12.08 mmol/L; range = 5.65–29.62 mmol/L; cutoff < 1.69 mmol/L). Dotted lines indicate the cutoff values for each parameter. Among these individuals, 14 out 20 (70.0%) presented an abnormal VLCFA profile. This included eight individuals with an increase in C26:0, one with an elevated C26:0/C22:0 ratio and five individuals with elevations in both C26:0 and C26:0/C22:0. In contrast, only one patient (H1) exhibited a slight increase in C24:0- and C26:0-LPC (0.30 and 0.23 μmol/L, respectively), and this patient also showed increases in C26:0 and the C26:0/C22:0 ratio.

As plasma VLCFA elevations can sometimes be secondary in individuals with hypertriglyceridemia, we conducted an analysis of both very long-chain LPCs and VLCFA in 20 plasma samples with elevated triglyceride concentrations ranging between 5.65 and 29.62 mmol/L (median = 12.08 mmol/L, cutoff < 1.69 mmol/L). Among these samples, 14 (70.0%) exhibited an increase in either C26:0 and/or the C26:0/C22:0 ratio. Specifically, eight samples presented elevated C26:0 levels, one sample showed an increase in the C26:0/C22:0 ratio, and five samples demonstrated an increase in both C26:0 and C26:0/C22:0 ratio. However, it is worth noting that only one of these samples exhibited a slight increase in C24:0- and C26:0-LPC (0.30 and 0.23 μmol/L, respectively). This particular sample also presented an increase of C26:0 and C26:0/C22:0 ratio (see Fig. 2).

### C26:0-LPC/C22:0-LPC ratio is useful to biochemically differentiate ALD from ZSD patients

Despite an overall low sensitivity for C22:0-LPC (75.0%), notably higher sensitivity was observed in ALD patients (89.0% for ALD males and 77.9% for ALD females) when compared with ZSD patients (44.3%) (Table 3). This discrepancy was explained by the differences in plasma C22:0-LPC concentrations, with ZSD patients exhibiting significantly lower levels (mean = 0.24 μmol/L, SD = 0.14 μmol/L) when compared with ALD males (mean = 0.40 μmol/L, SD = 0.19 μmol/L) (*P* < 0.0001) and ALD females (mean = 0.30 μmol/L, SD = 0.17 μmol/L) (*P* < 0.05). In contrast, plasma C26:0-LPC concentrations were significantly higher in ZSD patients (mean = 1.42 μmol/L, SD = 0.81 μmol/L) when compared with ALD males (mean = 0.64 μmol/L, SD = 0.27 μmol/L) (*P* < 0.0001) and ALD females (mean = 0.41 μmol/L, SD = 0.17 μmol/L) (*P* < 0.0001) (Table 2).

**Fig. 3:**
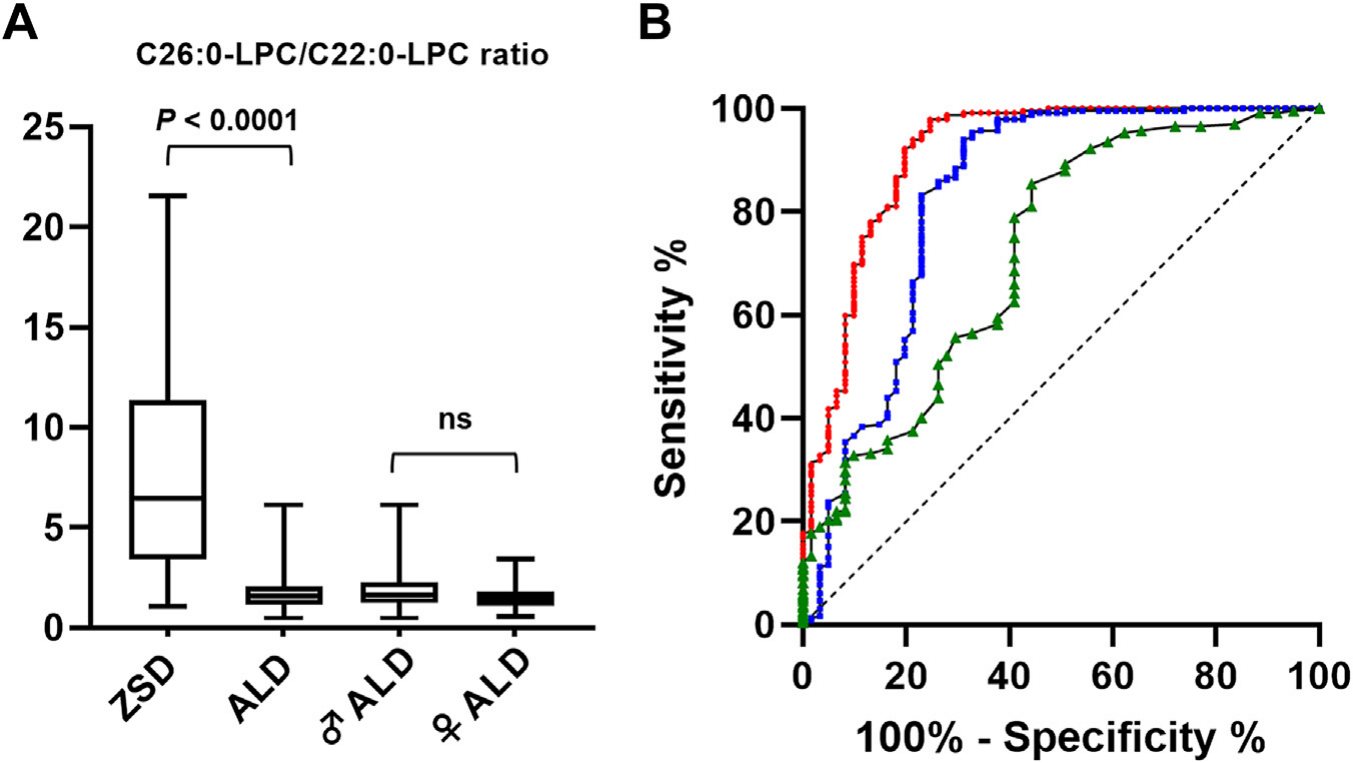
The C26:0-LPC/C22:0-LPC ratio is a valuable parameter to biochemically differentiate ZSD from ALD patients. A: The C26:0-LPC/C22:0-LPC ratios obtained for the different patient groups are shown. Abbreviations are as follows, with the number of patients in each group indicated in brackets: ZSD = Zellweger spectrum disorder patients (n = 61), ALD = total adrenoleukodystrophy patients including males and females (n = 232), ♂ ALD = male adrenoleukodystrophy patients (n = 155), ♀ ALD = female adrenoleukodystrophy patients (n = 77), ns = not statistically significant. Since differences in the C26:0-LPC/C22:0-LPC ratio between ALD males and ALD females were not statistically significant, all ALD individuals were considered as a single group for this analysis. B: ROC curve analysis demonstrated superior performance for the C26:0-LPC/C22:0-LPC ratio (red curve) to differentiate ZSD and ALD patients compared with individual plasma C26:0-LPC (blue curve) or C22:0-LPC (green curve) concentrations. The results of the ROC curve analyses are as follows (CI = confidence interval; AUC = area under the curve): C26:0-LPC/C22:0-LPC ratio: AUC = 0.91 (95% CI 0.86–0.96); C26:0-LPC: AUC = 0.83 (95% CI 0.76–0.90); C22:0-LPC: AUC = 0.72 (95% CI 0.64–0.80). ROC, receiver operating characteristic.

Subsequently, we evaluated the performance of the C26:0-LPC/C22:0-LPC ratio to biochemically differentiate ALD from ZSD patients. As no differences in C26:0-LPC/C22:0-LPC ratio were observed between ALD males and females (Fig. 3A), all ALD patients were considered as a single group for this purpose. Receiver operating characteristic curve analysis showed an area under the curve (AUC) of 0.91 (95% CI 0.86–0.96) for the C26:0-LPC/C22:0-LPC ratio, indicating a better performance compared with using individual concentrations of C26:0-LPC (AUC = 0.83; 95% CI 0.76–0.90) and C22:0-LPC (AUC = 0.72; 95% CI 0.64–0.80) to differentiate between disease groups (Fig. 3B). Ratio values below 1.07 gave a PPV of 100.0% for a diagnosis of ALD in our cohort, whereas ratio values greater than 6.15 resulted in a PPV of 100.0% for a ZSD diagnosis.

## Discussion

The diagnostic accuracy of using plasma concentrations of very long-chain LPCs in a large cohort was examined in this study. Our primary goal was to compare the diagnostic performance of plasma LPCs with VLCFA gold-standard analysis in individuals affected by peroxisomal β-oxidation disorders, including ALD and ZSD.

To achieve this, we developed a plasma very long-chain LPCs analysis method by adapting the NeoBase™ 2 Non-derivatized MSMS kit, originally designed for DBS samples. The validation of our method revealed its robust analytical performance across all three evaluated LPCs. We established populational cutoff values for C22:0-, C24:0-, and C26:0-LPC and assessed differences among the age groups. The observed differences did not reach clinical significance, enabling us to adopt the same cutoffs across all age groups. Regarding sex-based variations in very long-chain LPCs concentrations, our results indicated no statistically significant differences for any of the three LPCs, consistent with previous reports (35). Additionally, since hypertriglyceridemia can lead to secondary elevations in plasma levels of VLCFA (9), we analyzed both very long-chain LPCs and VLCFA in samples with high triglyceride concentrations. Interestingly, the majority (70.0%) of individuals exhibited an altered VLCFA profile, while only one sample showed an increase of C24:0- and C26:0-LPC. This suggests that plasma very long-chain LPCs may exhibit greater specificity as biomarkers in the presence of hypertriglyceridemia compared with VLCFA.

Concerning diagnostic performance, our data highlighted that C26:0-LPC exhibited the highest accuracy, with a sensitivity and a specificity of 98.8%. While our results were slightly lower than the diagnostic accuracy of 100% reported by Jaspers *et al.* (28), it is important to note that our study included a larger number of patients and controls, adding robustness to our findings. Moreover, our study ventured into the diagnostic performance of C22:0- and C24:0-LPC, which had not been investigated in plasma before. Impressively, C24:0-LPC demonstrated excellent diagnostic accuracy, with a sensitivity and a specificity of 98.5% and 98.3%, respectively. Conversely, C22:0-LPC exhibited a poor performance with only a sensitivity of 75.1%. Particularly noteworthy was that the highest diagnostic sensitivity was achieved by considering the combination of C24:0- and C26:0-LPC, correctly classifying 99.7% of all PD patients. In contrast, plasma VLCFA analysis, despite its notable performance with the combination of C24:0/C22:0 and C26:0/C22:0 ratios, exhibited a lower sensitivity. Specifically, five out 77 ALD females and one patient with Heimler syndrome displayed normal VLCFA concentrations and ratios, indicating a decreased sensitivity compared with the C24:0- and C26:0-LPC combination, which was normal in only a single ALD female.

In terms of false-negative outcomes, it is essential to highlight that our findings showcase significantly enhanced sensitivity for diagnosing ALD in females when utilizing C24:0- and C26:0-LPC, in contrast to VLCFA analysis. However, we are also reporting here the first case of an ALD female presenting with normal plasma very long-chain LPCs concentrations. This underscores the need for caution when interpreting LPC results in this subgroup, where normal concentrations may appear due to X-chromosome preferential inactivation, as is also the case in VLCFA analysis.

It is essential to acknowledge that the high sensitivity observed in ALD females for very long-chain LPCs analysis in our study may be explained by a patient selection bias favoring females with altered VLCFA profiles. This bias is substantiated by the fact that only 6.5% of females in our study exhibited normal plasma VLCFA concentrations, a notably lower percentage compared to previous reports where normal VLCFA levels were observed in 15%–31% of ALD females (4, 9). We want to highlight that previous reports analyzing C26:0-LPC in DBS samples for ALD newborn screening may also generate biased results with respect to ALD females, as they primarily detect individuals with biochemical alterations (9, 17, 18, 19, 20, 21, 22, 23, 24, 31). Additionally, as mentioned earlier, one Heimler syndrome patient tested negative for VLCFA analysis. Although this patient was correctly classified by LPCs analysis, we observed only a borderline increase in C24:0-LPC alongside a normal C26:0-LPC concentration. Hence, it remains plausible that individuals with the mildest phenotypes of the Zellweger spectrum could also exhibit normal very long-chain LPC concentrations, mirroring what is observed with VLCFA analysis (7). In line with this, Jaspers *et al.* 2020 also reported ALD female and ZSD patients presenting C26:0-LPC concentrations in DBS samples very close to the upper limit of the reference range (28). Consequently, it is essential to exercise the same level of caution used with VLCFA analysis when interpreting very long-chain LPCs results, particularly in these patient groups. Further studies involving larger and unbiased cohorts are required to comprehensively assess the diagnostic performance of very long-chain LPCs in both ALD females and ZSD patients presenting with mild phenotypes.

Finally, while C22:0-LPC analysis did not add any additional diagnostic power to our study, with C26:0-LPC and C24:0-LPC sufficient for accurate patient classification, our findings suggest an interesting avenue for differentiating between ALD and ZSD patients. Specifically, the C26:0-LPC/C22:0-LPC ratio may emerge as a valuable parameter. Even though the clinical utility of this parameter may be limited, as it only achieved certainty in cases with extreme values, this ratio holds the potential to offer a preliminary differential diagnosis, particularly in scenarios where comprehensive clinical data are not readily available to the laboratory.

In conclusion, this study demonstrates that the analysis of plasma very long-chain LPCs is a highly accurate and robust method for diagnosing peroxisomal β-oxidation disorders. We strongly recommend the integration of plasma C24:0- and C26:0-LPC as a first step in patients suspected of having a PD. The introduction of these novel biomarkers offers significant advantages over the gold-standard not only regarding the shortening and simplification of the analytical process (using LC-MS/MS) but also by demonstrating better diagnostic accuracy than with plasma VLCFA analysis, particularly in ALD females. Furthermore, the incorporation of C22:0-LPC analysis and the C26:0-LPC/C22:0-LPC ratio can serve as a tool to biochemically differentiate ALD from ZSD patients. Further studies involving larger and more diverse patient populations will be necessary to confirm and extend these findings, ultimately paving the way for improved and efficient diagnosis of these conditions.

## Data availability

All relevant data in this study are available in the manuscript, supplementary information, tables, and figures.

## Supplemental data

This article contains supplemental data.

## Conflict of interest

The author declares that they have no conflicts of interest with the contents of this article.
