# Supplementary material for: Plasma C24:0- and C26:0-lysophosphatidylcholines are reliable biomarkers for the diagnosis of peroxisomal β-oxidation disorders
Source: J Lipid Res. 2024 Feb 4;65(3):100516. doi: 10.1016/j.jlr.2024.100516 (PMC10910329; doi:10.1016/j.jlr.2024.100516)
Supplement: Supporting Information [file mmc1.docx]

**SUPPLEMENTAL DATA**

**Plasma C24:0- and C26:0-lysophosphatidylcholines are Reliable Biomarkers for the Diagnosis of Peroxisomal β-oxidation Disorders**

Blai Morales-Romero^1,2,3^, José Manuel González de Aledo-Castillo^1^, Cristina Fernández Sierra^1^, Carmen Martínez Carreira^1^, Carles Zaragoza Bonet^1^, Rosa Fernández Bonifacio^4^, Maria Antònia Caro Miró^1^, Ana Argudo-Ramírez^1^, Rosa María López Galera^1,2,3^, Judit García-Villoria^1,2,3^

^1^ Section of Inborn Errors of Metabolism-IBC, Biochemistry and Molecular Genetics Department, Hospital Clínic de Barcelona, Barcelona, Spain.

^2^ Biomedical Research Institute August Pi i Sunyer (IDIBAPS), Barcelona, Spain.

^3^ Center for Biomedical Research Network on Rare Diseases (CIBERER), Madrid, Spain.

^4^ CORE Laboratory, Biochemistry and Molecular Genetics Department, Hospital Clínic de Barcelona, Barcelona, Spain.

**
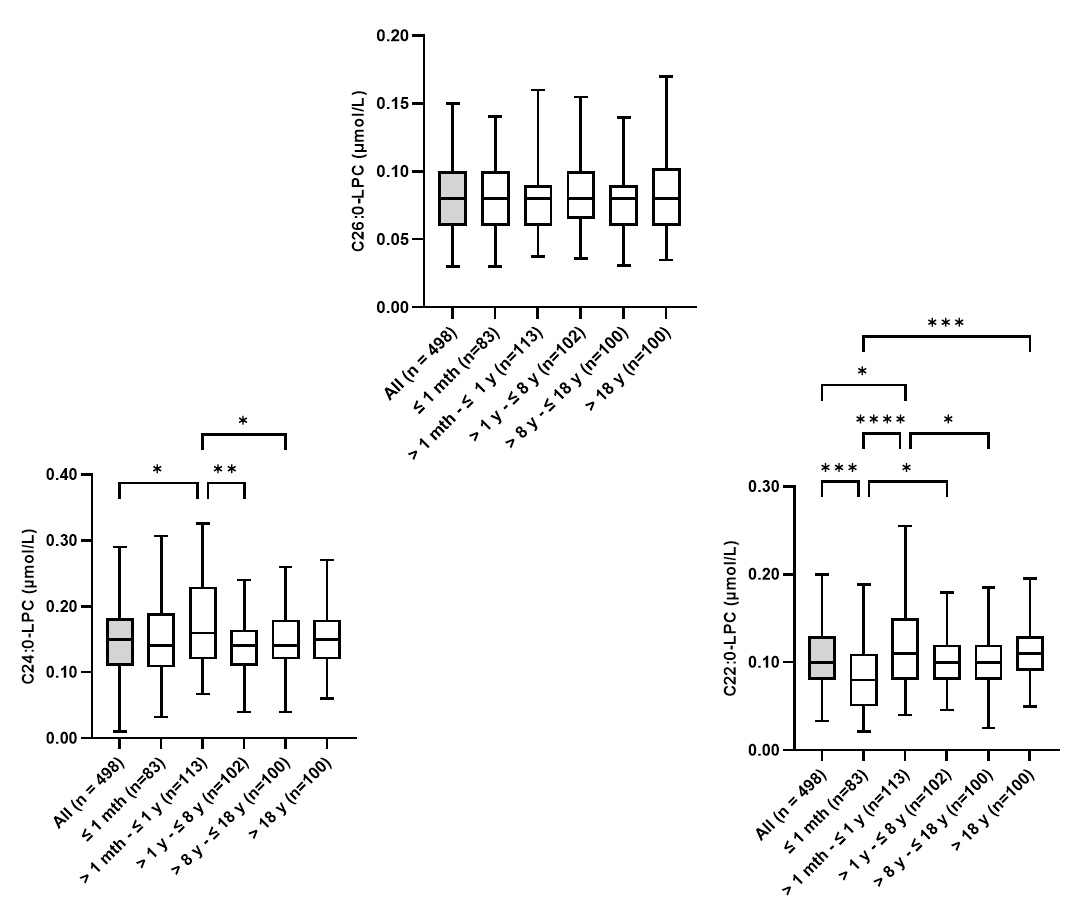
**

**Supplemental Figure S1**: **Plasma C22:0-, C24:0- and C26:0-LPC concentrations in different age groups.**

The results are depicted using box and whisker plots spanning from the 2.5^th^ and the 97.5^th^ percentiles. No statistically significant differences in plasma C26:0-LPC concentrations were observed across different age groups. However, for plasma C22:0- and C24:0-LPC, we found significantly higher concentrations in patients aged between one month and one year compared to other groups. Additionally, the plasma C22:0-LPC concentration in patients less than one month old was significantly lower compared to those aged one to eight years old and those older than 18 years old.

Abbreviations and levels of significance are as follows: mth = months; y = years; n = number of individuals. * (*P* ≤ 0.05); ** (*P* ≤ 0.01); *** (*P* ≤ 0.001); **** (*P* ≤ 0.0001); *P* = P-value.

**Supplemental** **Figure S2:** **Gender-specific variations assessment in plasma very long-chain LPC concentrations.**

No statistically significant differences were observed for plasma C22:0-, C24:0- and C26:0-LPC between males (n = 276) and females (n = 222). (C26:0-LPC: *P* = 0.979; C24:0-LPC: *P* = 0.582; C22:0-LPC: *P* = 0.948).

Abbreviations are as follows: n = number of individuals; *P* = P-value; ns = not statistically significant (*P* > 0.05).
